# Gemeinsame Grundlage von Alexithymie und expressiver Suppression: Ergebnisse einer empirischen Studie

**DOI:** 10.1007/s00278-021-00546-x

**Published:** 2021-10-27

**Authors:** Vera Schiewer, Thilo Dietz, Sally Tavenrath, Hülya Öztürk-Arenz, Reinhold S. Jäger, Anne Klein, Hildegard Labouvie, Michael Kusch

**Affiliations:** 1 Psychoonkologische Versorgungsforschung, Klinik I für Innere Medizin, Universitätsklinikum Köln, Köln, Deutschland; 2 Zentrum für Empirische Pädagogische Forschung, Universität Koblenz-Landau, Campus Landau, Landau, Deutschland

**Keywords:** Affektive Störungen, Emotionen, Faktorenanalyse, Sprachlosigkeit, Umfragen und Fragebogen, Affective symptoms, Emotions, Factor analysis, Speechlessness, Surveys and questionnaires

## Abstract

**Hintergrund:**

Internationale Studien konnten bereits einen Zusammenhang zwischen Alexithymie und expressiver Suppression belegen. Im deutschsprachigen Raum wurde dieser Zusammenhang bisher nur sehr selten betrachtet. Übergeordnetes Ziel der vorliegenden Studie war die Untersuchung eines korrelativen und faktoriellen Zusammenhangs von Alexithymie und expressiver Suppression.

**Material und Methoden:**

Insgesamt 317 Personen nahmen einer Onlinebefragung teil. Mithilfe der deutschsprachigen Versionen der Toronto Alexithymia Scale-26 (TAS-26) und des Emotion Regulation Questionnaire (ERQ) wurden Daten zu Alexithymie und expressiver Suppression erfasst.

**Ergebnisse:**

Es bestanden signifikante Korrelationen zwischen der Subskala „Schwierigkeit bei der Identifikation von Gefühlen“ der TAS-26 und der Skala „Unterdrückung“ des ERQ (r = 0,5; *p* < 0,001) sowie zwischen der Subskala „Schwierigkeit bei der Beschreibung von Gefühlen“ der TAS-26 und der Skala „Unterdrückung“ des ERQ (r = 0,64; *p* < 0,001). Die Ergebnisse einer explorativen Faktorenanalyse ergaben eine Zwei-Faktoren-Lösung mit einem gemeinsamen Faktor für die TAS-26-Subskalen „Schwierigkeit bei der Identifikation von Gefühlen“ und „Schwierigkeit bei der Beschreibung von Gefühlen“ und der Skala „Unterdrückung“ des ERQ mit einer gemeinsamen Varianz von 38,2 % (χ^2^ = 363,843; *p* < 0,001; Kaiser-Meyer-Olkin[KMO]-Wert = 0,699).

**Schlussfolgerung:**

Die Ergebnisse legen nahe, dass den Skalen der TAS-26 in den Komponenten „Schwierigkeit bei der Identifikation von Gefühlen“ und „Schwierigkeit bei der Beschreibung von Gefühlen“ sowie der Skala „Unterdrückung“ des ERQ in der Komponente der „expressiven Suppression“ ein gemeinsames Konstrukt zugrunde liegt, das mit dem Begriff der Sprachlosigkeit belegt werden kann.

## Abstract (en)

**Background:**

International studies could already prove a correlation between alexithymia and expressive suppression. This relationship has only been marginally considered in the German literature so far. The prioritized aim of the present study was to investigate a correlative and factorial relationship between alexithymia and expressive suppression.

**Material and methods:**

A total of 317 persons participated in an online survey. Data on alexithymia and expressive suppression were collected using the German versions of the Toronto alexithymia scale (TAS-26) and the emotion regulation questionnaire (ERQ).

**Results:**

The results showed highly significant correlations between the TAS-26 subscale “difficulty in identifying feelings” and the ERQ scale “suppression” (r = 0.5; *p* < 0.001) and between the TAS-26 subscale “difficulty in describing feelings” and the ERQ scale “suppression” (r = 0.64; *p* < 0.001). The results of an exploratory factor analysis revealed a two-factor solution with common factor for the TAS-26 subscales “difficulty in identifying feelings” and “difficulty in describing feelings” and the ERQ scale “suppression” with a common variance of 38.2% (χ^2^ = 363.843, *p* < 0.001, Kaiser-Meyer-Olkin, KMO, value = 0.699).

**Conclusion:**

The results indicate that the TAS-26 scales in the components “difficulty in identifying feelings” and “difficulty in describing feelings” and the ERQ scale “suppression” in the component of “expressive suppression” have a common construct, which is referred to with the term speechlessness.

Die Wahrnehmung der eigenen Emotionen und ihre Äußerung nach außen bilden ein zentrales Merkmal zwischenmenschlicher Kommunikation. Personen können sich jedoch in ihrer Fähigkeit, die eigenen inneren emotionalen Zustände wahrzunehmen und zu äußern, unterscheiden. Einen in der Literatur häufig genannten Grund für diese fehlende bis erschwerte Einsicht in die eigenen Emotionen bildet die Alexithymie. Die expressive Suppression wird hingegen genannt, wenn Personen ihre Emotionen nicht mitteilen. Ziel der Studie war es, einen Zusammenhang beider Konzepte an einer deutschen Stichprobe zu untersuchen.

## Alexithymie und expressive Suppression

### Krankheitsbilder und Zusammenhang

Personen mit einer Alexithymie oder geringer Emotionswahrnehmung zeigen wenige oder gar keine Fähigkeiten für innerlich stattfindende emotionale Prozesse (Gross 2014; Lindquist und Barrett 2008). Personen mit expressiver Suppression wiederum neigen dazu, den expressiven Ausdruck ihrer Emotionen trotz Zugang zum eigenen emotionalen Befinden bewusst zu unterdrücken (Gross 2014). Internationale Untersuchungen haben bislang gezeigt, dass Personen mit einer hohen Ausprägung an Alexithymie sowohl Schwierigkeiten bei der Identifikation und Beschreibung der eigenen Emotionen aufweisen als auch die eigenen Emotionen vermehrt bewusst unterdrücken können (Kessler et al. 2010; Laloyaux et al. 2015; Swart et al. 2009). Alexithymie und expressive Suppression können getrennt voneinander oder in Kombination auftreten (Laloyaux et al. 2015; Swart et al. 2009). Personen mit stark ausgeprägter Alexithymie können in hoch emotionalen Situationen auftretende physiologische Reaktionen (etwa feuchte Hände und eine erhöhte Herzfrequenz) nur schwer einer konkreten Emotion zuordnen (etwa als Zeichen von „Angst“) und/oder nur schwer adäquat beschreiben (Bagby et al. 2020; Taylor et al. 1997). Bei Personen ohne oder mit gering ausgeprägter Alexithymie kann in entsprechenden Situationen die Emotionsregulationsstrategie der expressiven Suppression vorliegen, in der physiologische Reaktionen zwar wahrgenommen und beispielsweise als Angst erlebt werden, der behaviorale Ausdruck jedoch bewusst unterdrückt wird (Gross 2014).

Die internationale Literatur bestätigte bereits einen Zusammenhang zwischen dem Konzept der Alexithymie und dem Konzept der expressiven Suppression als Teil der Regulation von Emotionen (Brandão et al. 2017; Erkic et al. 2018; Goerlich-Dobre et al. 2014; Kessler et al. 2010; Laloyaux et al. 2015; Olalde-Mathieu et al. 2021; Swart et al. 2009). Insbesondere belegten die Untersuchungen eine signifikante Korrelation zwischen der Schwierigkeit einer Person, Gefühle zu identifizieren und zu beschreiben, sowie der Unterdrückung der Äußerung von Emotionen (Brandão et al. 2017; Kessler et al. 2010; Laloyaux et al. 2015; Olalde-Mathieu et al. 2021; Swart et al. 2009). Inwiefern sich diese Studien für eine deutschsprachige Stichprobe replizieren lassen, wurde bislang nicht untersucht.

### Entwicklung und Validierung der Erfassungsinstrumente

Alexithymie beschreibt ein ursprünglich aus der Psychosomatik stammendes Konzept der Unfähigkeit, eigene Gefühle adäquat wahrzunehmen und sie mit Worten zu beschreiben (López-Muñoz und Pérez-Fernández 2020; Sifneos 1973; Taylor et al. 1991). Die Erfassung des psychologischen Konstrukts der Alexithymie erfolgte in den weitaus meisten Fällen unter Verwendung der Toronto Alexithymia Scale (Bagby et al. 2020; Gaggero et al. 2020). Der Fragebogen liegt in einer Version mit 26 (TAS-26; Taylor et al. 1985) sowie einer Version mit 20 Items (TAS-20; Bagby et al. 1994) vor. Die TAS-26 (Taylor et al. 1985) erfasst Alexithymie mithilfe folgender 4 Faktoren:

- „Difficulties Identifying Feelings“ (DIF, dt.: Schwierigkeit, die eigenen Gefühle zu identifizieren),
- „Difficulties Describing Feelings“ (DDF, dt.: Schwierigkeit, die eigenen Gefühle zu beschreiben),
- „Extern Oriented Thinking“ (EOT, dt.: extern orientierter Denkstil),
- „Difficulties Fantasising“ (DFAN, dt.: eingeschränktes Fantasieren aufgrund eines Mangels an Tagträumen).

Empirische Untersuchungen zum Konzept der Alexithymie konnten diese ursprüngliche Vier-Faktoren-Struktur der TAS-26 (Taylor et al. 1985) nicht bestätigen (Bagby et al. 1994). Daraufhin erfolgte die Weiterentwicklung zur TAS-20 (Bagby et al. 1994), einer gekürzte Version der TAS-26 (Taylor et al. 1985), mit einer Drei-Faktoren-Struktur ohne die DFAN-Komponente (Bagby et al. 2020).

Die Entwicklung und Validierung der deutschsprachigen Version verliefen von der TAS-20 zur TAS-26. Beginnend mit der TAS-20 erfolgte durch die Forschergruppe um Bach et al. (1996) eine Umsetzung und Prüfung des englischsprachigen Originals (Bagby et al. 1994). Diese Version erzielte jedoch nur eine geringe interne Konsistenz und geringe Faktorladungen. Entsprechend den Ergebnissen entwickelten und validierten Kupfer et al. (2001) die deutsche Version der TAS-26. Im Unterschied zum englischsprachigen Original wurden in der deutschen Version zwar 26 Items übersetzt, es werden jedoch ausschließlich 18 Items über die Subskalen DIF, DDF und EOT ausgewertet. Faktorenlösung und Teststatistik der deutschen Version der TAS-26 (Kupfer et al. 2001) erwiesen sich der ursprünglichen deutschsprachigen Fassung der TAS-20 (Bach et al. 1996) als deutlich überlegen.

Die expressive Suppression ist eine reaktionsfokussierte Regulationsstrategie, die sich auf die Unterdrückung des aktuellen Ausdrucks einer Emotion bezieht, nachdem sie hervorgerufen wurde. Die kognitive Neubewertung („cognitive reappraisal“) stellt dagegen eine Antezedens-fokussierte Regulationsstrategie dar, die eine emotionsauslösende Situation mit dem Ziel neu bewertet, ihren emotionale Auswirkung zu verändern (Gross 2014). Gross und Thompson (2006) betonen, dass die expressive Suppression im Wesentlichen eine Modulation auf Verhaltensebene einer emotionalen Reaktion ist, ohne das Erleben dieser Emotion zu reduzieren. Basierend auf der Theorie der Regulation von Emotionen von Gross (1998) wurde der Emotion Regulation Questionnaire (ERQ) zur Erfassung der emotionsregulierenden Strategien (1) der kognitiven Neubewertung mithilfe der Skala „reappraisal“ und (2) der expressiven Suppression mithilfe der Skala „suppression“ entwickelt (Gross und John 2003). Eine deutschsprachige Umsetzung des ERQ mit identischer Item-Zahl sowie einer gleichwertigen Reliabilität und Faktorenstruktur erfolgte durch Abler und Kessler (2009).

### Literaturrecherche zum Forschungsstand

Recherchen in den Datenbanken *PubMed, Web of Science* und *PsycInfo* unter Verwendung des einheitlichen Suchterms „{((TAS-26 german version) AND (ERQ)) AND (correlation)}“ ergaben keine Ergebnisse für Studien, die einen Zusammenhang zwischen Alexithymie und expressiver Suppression bzw. Unterdrückung unter Verwendung der deutschsprachigen Version der TAS-26 (Kupfer et al. 2001) und des ERQ (Abler und Kessler 2009) untersuchen. Es liegen vereinzelt Studien an deutschsprachigen Stichproben vor, die einen Zusammenhang zwischen Alexithymie und expressiver Suppression unter Verwendung der deutschsprachigen TAS-20 (Bach et al. 1996) analysieren (Erkic et al. 2018; Goerlich-Dobre et al. 2014; Kessler et al. 2010). Diese beinhalteten Patientengruppen mit M. Parkinson und somatoformen Störungen (Erkic et al. 2018). Fokus beider Studien bildet jedoch der Einfluss einer Erkrankung auf die Regulation von Emotionen und nicht der Zusammenhang zwischen Alexithymie und expressiver Suppression. Im deutschen Sprachraum prüfte allein die Studie von Kessler et al. (2010) einen direkten korrelativen und faktoriellen Zusammenhang zwischen den Konzepten Alexithymie und expressiver Suppression bzw. Unterdrückung unter Verwendung der deutschsprachigen TAS-20 (Bach et al. 1996) und des ERQ (Abler und Kessler 2009) an einer gesunden Stichprobe.

Der Studie von Kessler et al. (2010) liegt eine explorativ erhobene Drei-Faktoren-Struktur der TAS-20 zugrunde, die basierend auf Ergebnissen der Arbeit von Popp et al. (2008) konstruiert wurde. Entsprechend Kessler et al. (2010) änderte sich die ursprüngliche Skalenstruktur DIF, DDF und EOT des Fragebogens. Die Subskalen DIF und DDF wurden in der Skala „Erfassung der Schwierigkeit bei der Identifikation und Beschreibung von Gefühlen“ zusammengefasst, darüber hinaus erfolgten eine Änderung der EOT-Skala hin zur Skala „externaler Denkstil“ sowie die zusätzliche Konstruktion der Skala „Wichtigkeit emotionaler Introspektion“. Dadurch bedingt zeigen sich deutliche Unterschiede zwischen dem englischsprachigen Original der TAS-20 (Bagby et al. 1994) und der deutschsprachigen Version des Fragebogens (Bach et al. 1996; Kessler et al. 2010; Popp et al. 2008), was einen direkten Vergleich von deutschsprachigen und internationalen Studien nur eingeschränkt möglich macht.

Kessler et al. (2010) haben mit ihrer Studie als Erste einen Beitrag zur Untersuchung eines Zusammenhangs zwischen Alexithymie und expressiver Suppression geleistet.

## Ziel der Arbeit

Die vorliegende Arbeit nutzt die Systematik der Studie der Autoren, jedoch unter Verwendung der deutschsprachigen TAS-26 (Kupfer et al. 2001) und des deutschsprachigen ERQ (Abler und Kessler 2009) und mit einem ausschließlichen Fokus auf dem explorativen Zusammenhang mit der expressiven Suppression. Zusätzlich soll geprüft werden, ob mit höheren Ausprägungen der Alexithymie höhere Ausprägungen in der expressiven Suppression einhergehen.

## Methoden

Mithilfe einer Onlineerhebung konnten insgesamt 317 Personen zur Teilnahme bewegt werden. Dabei wurden Studierende der Universität zu Köln sowie Mitarbeitende der Universität und der Uniklinik Köln per E‑Mail-Verteiler kontaktiert und zur Teilnahme an der Erhebung gebeten. Insgesamt 81,4 % (*n* = 258) der Teilnehmenden waren weiblich. Das Durchschnittsalter betrug 35,8 Jahre (SD ± 13,04 Jahre; Spannweite: 18 bis 70 Jahre). Mit 52,4 % (*n* = 166) hatte ein Großteil der Teilnehmenden einen Hochschulabschluss.

### Emotion Regulation Questionnaire

Die deutschsprachige Fassung des ERQ (Abler und Kessler 2009) bildet ein validiertes Selbsterhebungsinstrument zur Erfassung der Regulation von Emotionen mithilfe von 10 Items auf einer 7‑stufigen Likert-Skala. Die Skalen Unterdrückung (bestehend aus 4 Items) und Neubewertung (bestehend aus 6 Items) des ERQ messen 2 Strategien der Regulation von Emotionen, entsprechend der Theorie der Regulation von Emotionen nach Gross (1998, 2014). Die interne Konsistenz wurde von den Autoren für die Unterdrückung mit einem Cronbachs α von 0,74 und die der Skala Neubewertung mit einem Cronbachs α von 0,76 angegeben (Abler und Kessler 2009).

### Toronto-Alexithymie-Skala-26

Die deutsche Version der Toronto Alexithymia Scale-26 (Kupfer et al. 2001) umfasst 26 Items auf einer 5‑stufigen Likert-Skala. Die Auswertung der Items erfolgt in 3 Subskalen, entsprechend dem Toronto-Modell der Alexithymie (Taylor et al. 1997): (1) DIF, (7 Items), (2) DDF (5 Items) und (3) EOT (6 Items) sowie einer Gesamtskala „Alexithymie“, bestehend aus den 3 Subskalen.

Die Autoren geben für ihre DIF-Subskala ein Cronbachs α von 0,84, für ihre DDF-Subskala ein Cronbachs α von 0,69 und für ihre EOT-Subskala ein Cronbachs α von 0,67 an. Die Gesamtskala bewerten sie mit einem Cronbachs α von 0,81 (Kupfer et al. 2001). Untersuchungen des Zusammenhangs wiesen auf eine starke Korrelation der Subskalen DIF und DDF (r = 0,57, *p* < 0,001) hin. Für die EOT-Subskala finden sich nur schwache und nichtsignifikante Korrelationen mit den Subskalen der DIF und der DDF (EOT und DIF: r = 0,06; EOT und DDF: r = 0,15; Kupfer et al. 2001).

In Anlehnung an Taylor et al. (1997) bezeichnen die Autoren der deutschen Version der TAS-26 (Kupfer et al. 2001) Personen ab einem Gesamtwert ≥54 als alexithym. Dieser Schwellenwert wurde auch zur Berechnung der Differenzierung in hoch und gering ausgeprägte alexithyme Teilnehmende der vorliegenden Stichprobe verwendet.

### Statistische Analyse

Zur statistischen Analyse wurde das Programm IBM SPSS Version 26 angewendet. Zusammenhänge wurden mithilfe der Pearson-Produkt-Moment-Korrelationen berechnet. Untersuchungen von Mittelwertsunterschieden hoch und gering ausgeprägter alexithymer Teilnehmender erfolgten mithilfe des *t*-Tests bei unabhängigen Stichproben. Die Berechnung einer explorativen Faktorenanalyse (EFA) erfolgte als Hauptachsen-Faktorenanalyse mit der Varimax-Rotation. Zur Extraktion wurde ein Eigenwert von 1 verwendet.

## Ergebnisse

Die Mittelwerte und Standardabweichung der Skalen der TAS-26 (Kupfer et al. 2001) und des ERQ (Abler und Kessler 2009) zeigt Tab. 1. Die durchschnittliche Ausprägung der Alexithymie-Gesamtskala betrug M = 46,51 (SD ± 9,62; Range = 24–79).

**Table 1:** 

Skala	Minimum	Maximum	Mittelwert	± Standardabweichung
*Toronto-Alexithymie-Skala-26 (TAS-26)*				
DIF	7	35	14,01	± 5,56
DDF	6	25	12,97	± 3,60
EOT	6	30	19,53	± 5,28
Alexithymie-Gesamtskala	24	79	46,51	± 9,62
*Emotion Regulation Questionnaire (ERQ)*				
Neubewertung	1	7	4,4	± 1,13
Unterdrückung	1	7	3,33	± 1,35

*n* = 317

*DDF* Schwierigkeit bei der Beschreibung von Gefühlen, *DIF* Schwierigkeit bei der Identifikation von Gefühlen, *EOT* extern orientierter Denkstil

Die Ergebnisse der Pearson-Produkt-Moment-Korrelationen finden sich in Tab. 2. Es zeigte sich ein starker Zusammenhang (Cohen 1992) zwischen der Subskala DIF und der Skala „Unterdrückung“ (r = 0,5 p < 0,001) sowie zwischen der Subskala DDF und der Skala „Unterdrückung“ (r = 0,642; *p* < 0,001). Zwischen der Emotionsregulationsstrategie der kognitiven Neubewertung und Alexithymie (ausgedrückt durch die Gesamtskala) stellte sich ein schwacher, signifikant negativer Zusammenhang dar.

**Table 2:** 

*Skala*	*Toronto-Alexithymie-Skala-26 (TAS-26)*				*Emotion Regulation Questionnaire (ERQ)*		
	*DIF*	*DDF*	*EOT*	*Alexithymie-Gesamtskala*	*Neubewertung*	*Unterdrückung*	
*Toronto-Alexithymie-Skala-26 (TAS-26)*							
*DIF*	1	0,614***	−0,051	0,780***	−0,287***	0,496***	
*DDF*	0,614***	1	−0,019	0,719***	−0,253***	0,642***	
*EOT*	−0,051	−0,019	1	0,512***	0,137*	−0,081	
*Alexithymie-Gesamtskala*	0,780***	0,719***	0,512***	1	−0,185***	0,482***	
*Emotion Regulation Questionnaire (ERQ)*							
*Neubewertung*	−0,287***	−0,253***	0,137*	−0,185***	1	−0,147***	
*Unterdrückung*	0,496***	0,642***	−0,081	0,482***	−0,147***	1	

*n* = 317

*DDF* Schwierigkeit bei der Beschreibung von Gefühlen, *DIF* Schwierigkeit bei der Identifikation von Gefühlen, *EOT* extern orientierter Denkstil

*p < 0,05; **p < 0,01; ***p < 0,001

Die Ergebnisse des *t*-Tests zeigten einen signifikanten Mittelwertsunterschied auf allen Skalen der TAS-26 (Kupfer et al. 2001) und des ERQ (Abler und Kessler 2009) zwischen der Gruppe Teilnehmender mit hoch und gering ausgeprägter Alexithymie. Hoch ausgeprägte alexithyme Teilnehmende wiesen deutlich erhöhte Skalenwerte der TAS-26 (Kupfer et al. 2001) und der Skala „Unterdrückung“ des ERQ (Abler und Kessler 2009) auf. Ausschließlich gering ausgeprägte alexithyme Teilnehmende erzielten höhere Mittelwerte auf der Skala „Neubewertung“ des ERQ (Abler und Kessler 2009; Tab. 3).

**Table 3:** 

Skala	M_gering_	± SD_gering_	M_hoch_	± SD_hoch_	*t*-Wert	Effektstärke
*Toronto-Alexithymie-Skala-26 (TAS-26)*						
DIF	12,00	± 3,82	20,88	± 5,06	−8,879***	0,67
DDF	11,89	± 3,00	16,63	± 3,02	−4,735***	0,26
EOT	18,68	± 5,37	22,42	± 3,73	−3,735***	0,28
Gesamtskala	42,57	± 6,57	59,92	± 5,28	−17,349***	0,82
*Emotion Regulation Questionnaire (ERQ)*						
Neubewertung	4,48	± 1,09	4,13	± 1,21	0,3511*	0,02
Unterdrückung	3,00	± 1,21	4,47	± 1,20	−1,470***	0,08

*n* = 317

*DDF* Schwierigkeit bei der Beschreibung von Gefühlen, *DIF* Schwierigkeit bei der Identifikation von Gefühlen, *EOT* extern orientierter Denkstil

*p < 0,05; **p < 0,01; ***p < 0,001

**Figure 1:**
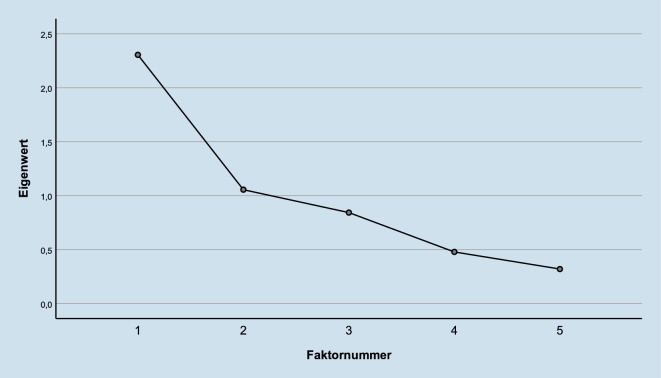

Die Erfassung latenter Strukturen zwischen Skalen der TAS-26 (Kupfer et al. 2001) und des ERQ (Abler und Kessler 2009) erfolgte mithilfe einer EFA. Barlett-Test {(χ^2^ (10)) = 363,843; *p* < 0,001)} und Kaiser-Meyer-Olkin-Kriterium {(KMO-Wert = 0,699)} weisen auf eine Eignung der Variablen für eine Faktorenanalyse hin. Anhand des Screeplots (Abb. 1) lässt sich erkennen, dass maximal 2 Faktoren einen Eigenwert >1 erzielen. Insgesamt erklären die beiden extrahierten Faktoren 46 % der Varianz (Tab. 4). Insgesamt wurden 3 Iterationen zur Faktorenrotation benötigt.

**Table 4:** 

Skala	Ladung auf Faktor		Kommunalität
	1	2	
Varianzaufklärung (%)	38,2	7,8	–
*Toronto-Alexithymie-Skala-26 (TAS-26)*			
DIF	0,661	−0,265	0,507
DDF	0,874	−0,149	0,786
EOT	−0,025	0,192	0,038
*Emotion Regulation Questionnaire (ERQ)*			
Neubewertung	−0,168	0,645	0,445
Unterdrückung	**0,723**	−0,065	0,527

*n* = 317

Hauptachsen-Faktorenanalyse, Varimax-rotiert, Eigenwert >1

*DDF* Schwierigkeit bei der Beschreibung von Gefühlen, *DIF* Schwierigkeit bei der Identifikation von Gefühlen, *EOT* extern orientierter Denkstil

Die Tab. 4 zeigt die Ergebnisse der rotierten Faktoren. Die Subskalen DIF, DDF und die Skala Unterdrückung, die eine hohe Korrelation untereinander aufweisen (Tab. 3), lagen mit hohen Werten auf Faktor 1. Die Subskala EOT (TAS-26) und die Neubewertung-Skala (ERQ) grenzten sich negativ vom Faktor 1 ab und erzielten positive Werte auf Faktor 2. Während die Skala Neubewertung eine hohe Faktorladung erzielt (0,645), ist die Faktorladung der EOT-Subskala (0,192) zu vernachlässigen.

In Tab. 5 sind Korrelationsergebnisse bisheriger internationaler Studien aufgeführt. Ein Vergleich der Ergebnisse ergab eine stärkere Korrelation der Subskalen DIF und der Skala Unterdrückung sowie der Subskala DDF und der Skala Unterdrückung in der vorliegenden Stichprobe. Die Ergebnisse der internationalen Studien und der vorliegenden Stichprobe weisen auf eine signifikant schwache bis nichtvorhandene Korrelation zwischen den Skalen der TAS-26 (Kupfer et al. 2001) und der Skala Neubewertung des ERQ (Abler und Kessler 2009) bzw. den Skalen der TAS-20 (Bach et al. 1996; Bagby et al. 1994; Olalde-Mathieu et al. 2021) und der Skala Neubewertung des ERQ (Abler und Kessler 2009; Gross und John 2003) hin (TAS-20: DIF und Neubewertung: −0,09–−0,4; DDF und Neubewertung: −0,3–0,02; TAS-26: DIF und Neubewertung: −0,287; DDF und Neubewertung: −0,185).

**Table 5:** 

Internationale Studien									Stichprobe			
Autor	Jahr	TAS-20-Version	ERQ-Version	Stichprobengröße (*n*)	Korrelation (TAS-20)				Stichprobengröße (*n*)	Korrelation (TAS-26, dt. Version)		
					DIF + DDF und UD (NB)	DIF und UD (NB)	DDF und UD (NB)	TAS-20, Gesamtscore und UD (NB)		DIF und UD (NB)	DDF und UD (NB)	TAS-26-Gesamtscore und UD (NB)
Schiewer et al. (vorliegende Arbeit)	2021	–	Deutsch	–	–	–	–	–	317	0,5*** (−0,287***)	0,64*** (−0,253***)	0,48*** (−0,185***)
Brandão et al. (2017)	2017	Englisch	Englisch	204	– (–)	0,31*** (−0,11)	0,41*** (0,02)	0,44*** (−0,8)	–	–	–	–
Laloyaux et al. (2015)	2015	Englisch	Englisch	255	– (–)	0,21** (−0,11)	0,50*** (−0,04)	0,40*** (−0,05)	–	–	–	–
Erkic et al. (2018)	2017	–^a^	Deutsch	70	– (–)	0,32*** (−0,4***)	0,56*** (−0,3**)	0,50*** (−0,44***)	–	–	–	–
Goerlich-Dobre et al. (2014)	2014	Englisch	Englisch	91	– (–)	0,21* (–)	0,34*** (–)	0,38*** (–)	–	–	–	–
Olalde-Mathieu et al. (2021)	2021	Spanisch	Spanisch	699	– (–)	0,37*** (−0,09**)	0,47*** (−0,11**)	0,45*** (−0,14***)	–	–	–	–
Kessler et al. (2010)	2010	Deutsch^b^	Deutsch	116	0,37** (−0,04)	– (–)	– (–)	– (–)	–	–	–	–

*DDF* „difficulties describing feelings“ (Schwierigkeit bei der Beschreibung von Gefühlen), *DIF* „difficulties identifying feelings“ (Schwierigkeit bei der Identifikation von Gefühlen), *ERQ* Emotion Regulation Questionnaire, *NB* Neubewertung, *TAS* Toronto-Alexithymie-Skala* UD* Unterdrückung

*p < 0,05; **p < 0,01; ***p < 0,001

^a^Es finden sich keine genauen Angaben dazu, welche Version der TAS-20 verwendet wurde

^b^Faktorenstruktur nach Popp et al. (2008)

## Diskussion

### Interpretation der Ergebnisse

#### Ziel der Arbeit

Primäres Ziel der vorliegenden Studie war die Replikation des aktuellen Forschungsstands zum Zusammenhang zwischen Alexithymie und expressiver Suppression an einer deutschen Stichprobe. Unter Verwendung der englischsprachigen TAS-20 (Bagby et al. 1994) und ERQ (Gross und John 2003) wurde international bereits mehrfach ein Zusammenhang zwischen den Konzepten der Alexithymie und der expressiven Suppression untersucht und bestätigt (Brandão et al. 2017; Erkic et al. 2018; Goerlich-Dobre et al. 2014; Kessler et al. 2010; Laloyaux et al. 2015; Olalde-Mathieu et al. 2021; Swart et al. 2009). Für den deutschsprachigen Raum geschah dies bisher ebenfalls unter Verwendung der ins Deutsche überführten Version der TAS-20 (Bach et al. 1996). Die vorliegende Arbeit bildet eine Erweiterung und Ergänzung des Erkenntnisstandes, da der Zusammenhang hier erstmals unter Verwendung der deutschsprachigen TAS-26 (Kupfer et al. 2001) untersucht wurde.

#### Positive signifikante Korrelationen zwischen TAS-16 und ERQ, Literaturvergleich

Die Ergebnisse zeigten einen positiven, signifikanten Zusammenhang zwischen der Subskala DIF und der Skala „Unterdrückung“ sowie zwischen der Subskala DDF und der Skala „Unterdrückung“. Personen mit einem Defizit in der Gefühlsidentifikation und -beschreibung tendierten vermehrt zur Unterdrückung im Sinne der expressiven Suppression. Ein signifikant positiver Zusammenhang zwischen den Subskalen DIF, DDF, der Alexithymie-Gesamtskala der deutschsprachigen TAS-26-Version (Kupfer et al. 2001) und der Skala Unterdrückung des deutschsprachigen ERQ (Abler und Kessler 2009) konnte belegt werden (Tab. 5). Die Ergebnisse bestätigen somit die Resultate vorausgegangener Studien (Brandão et al. 2017; Erkic et al. 2018; Goerlich-Dobre et al. 2014; Laloyaux et al. 2015; Olalde-Mathieu et al. 2021). Die Stichprobengröße der vorliegenden Studie überstieg diejenige internationaler Studien mit Ausnahme der von Olalde-Mathieu et al. (2021). Zusätzlich zeigte sich ein stärkerer korrelativer Zusammenhang (|r| = 0,5; *p* < 0,001) zwischen der Subskalen DIF und der Skala Unterdrückung sowie zwischen DDF und Unterdrückung der TAS-26 (Kupfer et al. 2001) und des ERQ (Abler und Kessler 2009) als der in internationalen Studien nachgewiesene (Brandão et al. 2017; Erkic et al. 2018; Goerlich-Dobre et al. 2014; Kessler et al. 2010; Laloyaux et al. 2015; Olalde-Mathieu et al. 2021).

#### Negative Korrelation zwischen TAS-16 und ERQ, Literaturvergleich

Diese Untersuchung konnte zudem zeigen, dass die Emotionsregulationsstrategie der kognitiven Neubewertung in einem negativen Zusammenhang zur Schwierigkeit einer Person steht, die eigenen Gefühle zu identifizieren und zu beschreiben. Dieser Aspekt wurde bereits durch Swart et al. (2009) und Laloyaux et al. (2015) bestätigt, kann jedoch nun auch unter Verwendung der TAS-26 (Kupfer et al. 2001) an einer deutschsprachigen Stichprobe verifiziert werden. So zeigte die Gruppe gering ausgeprägter alexithymer Teilnehmender eine signifikant geringere Tendenz in der Anwendung expressiver Suppression bzw. Unterdrückung als die der hoch ausgeprägten alexithymen Teilnehmenden (Tab. 3). Entgegengesetzt wiesen hoch ausgeprägte alexithyme Personen eine geringere Tendenz der Neubewertung auf (Tab. 3; gering ausgeprägt Alexithymie: M = 4,48 vs. hoch ausgeprägt Alexithymie: M = 4,13; *p* < 0,05). Untersuchungen an Stichproben von Patientinnen und Patienten mit physischen Erkrankungen weisen ebenso darauf hin, dass bei Personen mit stärker ausgeprägter Alexithymie eine verstärkte expressive Suppression bzw. Unterdrückung vorliegt (Guimond et al. 2020; Messina et al. 2011).

#### Weitere Vergleiche mit Studienergebnissen

Mit Bezug auf die in der Einleitung diskutierte, von Popp et al. (2008) und Kessler et al. (2010) verwendete Faktorenstruktur, ist ein direkter Vergleich der Ergebnisse der vorliegenden Arbeit mit denen von Kessler et al. (2010) nur bedingt möglich. Die in der Arbeit der Autoren zu einer Skala („Schwierigkeit bei der Identifikation und Beschreibung von Gefühlen“) zusammengefassten Subskalen DIF und DDF der TAS-20 (Bach et al. 1996) zeigen einen positiven Zusammenhang mit der Skala Unterdrückung des ERQ (Abler und Kessler 2009; Kessler et al. 2010). Dieser Zusammenhang kann mit der vorliegenden Arbeit auch für beiden getrennten Skalen der TAS-26 (Kupfer et al. 2001) bestätigt werden (Tab. 4). Sowohl zwischen der Subskala DIF und der Skala Unterdrückung als auch der Subskala DDF und der Skala Unterdrückung der deutschsprachigen TAS-26 (Kupfer et al. 2001) konnte in der vorliegenden Arbeit ein starker Zusammenhang gefunden werden. Dabei zeigte sich eine stärkere Korrelation als von Kessler et al. (2010) nachgewiesen (Tab. 5; DIF + DDF und Unterdrückung: r = 0,37; p < 0,01 vs. DIF und Unterdrückung: r = 0,5; p < 0,001 bzw. DDF und Unterdrückung: r = 0,64; *p* < 0,001).

Bezogen auf die faktorielle Struktur kann bestätigt werden, dass dem deutschsprachigen ERQ (Abler und Kessler 2009) eine zweifaktorielle Struktur zugrunde liegt (Abler und Kessler 2009; Kessler et al. 2010). Diese Ergebnisse reihen sich in die bereits international gut bestätigte Zwei-Faktoren-Struktur des ERQ ein (Brandão et al. 2017; Gross und John 2003). Die in der vorliegenden Studie explorativ ermittelte Zwei-Faktoren-Struktur kann die von Kupfer et al. (2001) vorgefundene Drei-Faktoren-Struktur der TAS-26 (Kupfer et al. 2001) nicht bestätigen. Darüber hinaus weist die Analyse darauf hin, dass die Subskalen DIF und DDF auf einem gemeinsamen Faktor laden (Faktor 1; Tab. 4), von dem die EOT-Subskala (Faktor 2; Tab. 4) abgegrenzt werden kann.

Internationale Studien haben sich bisher nicht mit der Untersuchung des faktoriellen Zusammenhangs der Konzepte Alexithymie und expressiver Suppression befasst. Nach Wissen der Autoren betrachtet allein die Arbeit von Kessler et al. (2010) diesen Aspekt. Kessler et al. (2010) konnte einen einfaktoriellen Zusammenhang von Alexithymie und expressiver Suppression unter Verwendung der deutschsprachigen Version der TAS-20 (Bach et al. 1996) und des ERQ (Abler und Kessler 2009) nachweisen. Die vorliegende Arbeit kann diesen Zusammenhang unter Verwendung der deutschsprachigen TAS-26 (Kupfer et al. 2001) replizieren.

#### Sprachlosigkeit als gemeinsames Merkmal von Alexithymie und expressiver Suppression

Die im Rahmen der Datenanalyse nachgewiesenen hohen Faktorladungen zwischen den Subskalen DIF, DDF der TAS-26 (Kupfer et al. 2001) und der Skala Unterdrückung des ERQ (Abler und Kessler 2009) mit einer Varianz von 38,2 % (Tab. 4) weisen darauf hin, dass die beiden Konzepte Alexithymie und expressive Suppression bzw. Unterdrückung gemeinsam einen großen Teil der Gesamtvarianz eines übergeordneten Konstrukts erklären können. Dabei bilden die Subskalen DIF und DDF der TAS-26 (Kupfer et al. 2001) die Komponenten des Toronto-Modells der Alexithymie (Taylor et al. 1997) und die Skala Unterdrückung des ERQ (Abler und Kessler 2009) die Emotionsregulationsstrategie der expressiven Suppression (Gross 1998, 2014) ab. Dies wirft die Frage auf, was das gemeinsame Merkmal von Alexithymie und expressiver Suppression ist. Nach Ansicht der Autoren könnte dieses gemeinsame Merkmal mit dem Begriff der Sprachlosigkeit in Anlehnung an Berger (2016) beschrieben werden. Berger (2016) versteht unter „Sprachlosigkeit“ („speechlessness“) eine willentliche oder unwillentliche, zeitliche begrenzte Reaktion, die durch hoch emotionale Situationen, eine verletzte Erwartungshaltung oder fehlendes Wissen bedingt sein kann. Der „Sprachlosigkeit“ könnte ein Defizit, eine Verdrängung oder Abwehr zugrunde liegen, wie es im Zusammenhang mit dem Persönlichkeitsmodell der Alexithymie diskutiert wird (Taylor et al. 2016) oder eine Vermeidung der Aufmerksamkeitslenkung, entsprechend dem Ansatz des Attention-Appraisal Model der Alexithymie (Preece et al. 2017). Ebenso könnte vermutet werden, dass die „Sprachlosigkeit“ Ausdruck einer emotionalen Dysregulation in emotional belastenden Situationen ist und die Merkmale einer Alexithymie nur vorübergehend vorliegen oder ähnlich ein zeitstabiles Persönlichkeitsmerkmal bilden (Laloyaux et al. 2015; Martínez-Sánchez et al. 2003; Preece et al. 2020).

Ergänzend zur Alexithymie kann die affective agnosia (dt.: affektive Agnosie) (Lane et al. 2015) herangezogen werden. Diese wurde in der Arbeit von Kessler et al. (2010) in Form der Levels of Emotional Awareness Scale (LEAS; Lane et al. 1990) aufgegriffen, inhaltlich jedoch nicht unter Gesichtspunkten der affektiven Agnosie diskutiert. Der LEAS zeigt aufgrund seiner Systematik als Erhebungsinstrument ohne Item-Formulierung mit mehrstufigen Antwortskalen geringe Korrelationen mit der TAS (Kessler et al. 2010; Maroti et al. 2018). Die Autorengruppe um Lane (Lane et al. 2015) bezeichnen Affective agnosia als eine schwere Form der Alexithymie und führen diese weniger auf fehlende Worte für Emotionen als vielmehr auf eine fehlende Wahrnehmung der Emotionen aufgrund einer nicht stattfindenden Aktivierung des Wahrnehmungsprozesses bzw. einer fehlender Kompetenz der Wahrnehmung zurück (Smith et al. 2019). Dabei stimmen die Autoren mit dem Toronto-Modell von Taylor et al. (1997) in den Punkten eines Defizits in der Emotionswahrnehmung überein, führen dieses jedoch nicht auf ein reines Fehlen der Worte hin zu einer fehlenden kognitiven Wahrnehmung dieser zurück (Lane et al. 2015; Smith et al. 2019). Die Affective agnosia bildet einen weiteren Standpunkt in der Debatte um die Alexithymie als situationsabhängiges oder zeitstabiles Persönlichkeitsmerkmal (Laloyaux et al. 2015; Martínez-Sánchez et al. 2003; Preece et al. 2020) und bekräftigt die Vermutung eines übergreifenden bzw. zugrunde liegenden Konstrukts, das mit dem Begriff der „Sprachlosigkeit“ bezeichnet werden kann. Es bedarf jedoch weiterer Studien, um ein potenziell zugrunde liegendes Konstrukt der „Sprachlosigkeit“ näher zu bestimmen. Erste Ansätze wären eine theoretische Auseinandersetzung mit dem Begriff der „Sprachlosigkeit“, um diesen in den wissenschaftlichen Kontext einzuordnen, sowie die Konstruktion eines Erhebungsinstrumentes zur empirischen Erfassung von Sprachlosigkeit.

### Limitationen der Studie

Die Ergebnisse der vorliegenden Studie unterliegen einigen Limitationen. Bedingt durch Restriktionen aufgrund der durch das „severe acute respiratory syndrome coronavirus 2“ (SARS-CoV-2) ausgelösten Pandemie war die Durchführung üblicher Erhebungsmethoden (beispielsweise die Randomisierung der Stichprobe) nicht möglich. Entsprechend musste auf eine alternative Form der Datenerhebung mithilfe von Online-Umfragen zurückgegriffen werden. Bedingt durch diese Methodik können die Selbstselektion der Teilnehmenden und eine daraus potenziell resultierende Verzerrung der Ergebnisse nicht ausgeschlossen werden. Auch erfolgte keine Unterscheidung hinsichtlich physischer oder psychischer Beeinträchtigung der Teilnehmenden, da entsprechende Informationen nicht erhoben wurden. Die Skalenmittelwerte können daher nicht generalisiert werden. Des Weiteren zeigten sich eine ungleiche Geschlechterverteilung und fehlende Normalverteilung, was bei der Betrachtung der Ergebnisse berücksichtigt werden sollte. Ebenfalls sollte in Betracht gezogen werden, dass Studierende und Mitarbeiter aufgrund der Pandemie unter einer andauernden physischen und/oder psychischen Belastung stehen und dadurch das Antwortverhalten ebenfalls verzerrt sein könnte.

Ergänzend ist zu betrachten, dass es sich bei der originalen und deutschen Version der TAS (Bach et al. 1996; Bagby et al. 1994; Kupfer et al. 2001) und des ERQ (Abler und Kessler 2009) um Selbsterhebungsinstrumente mit Fragestellungen in Item-Form und Antwortmöglichkeiten auf einer mehrstufigen Likert-Skala handelt. Alternative Erhebungsformen wie die LEAS (Lane et al. 1990) ermöglichen aufgrund ihrer Erhebungsstruktur keine Verzerrung der Ergebnisse durch die soziale Erwünschtheit der befragten Person. Diese strukturellen Unterschiede in der Erhebungsform spiegeln sich auch in korrelativen Ergebnissen beider Instrumente wider (Maroti et al. 2018). Eine introspektive und extrospektive Betrachtung von Emotionen ähnlich der Form des LEAS (Lane et al. 1990) sollte berücksichtigt werden.

## Fazit

- Es besteht ein korrelativer und faktorieller Zusammenhang zwischen Alexithymie und expressiver Suppression.
- Die gemeinsame Grundlage beider psychologischer Konzepte kann mit dem Begriff der Sprachlosigkeit bezeichnet werden.
- Weitere Untersuchungen zu Sprachlosigkeit und einem potenziellen Kausalzusammenhang zwischen Alexithymie und expressiver Suppression sind notwendig.
